# Insights into the Molecular Structure, Stability, and Biological Significance of Non-Canonical DNA Forms, with a Focus on G-Quadruplexes and i-Motifs

**DOI:** 10.3390/molecules29194683

**Published:** 2024-10-02

**Authors:** Patrycja Obara, Paweł Wolski, Tomasz Pańczyk

**Affiliations:** Jerzy Haber Institute of Catalysis and Surface Chemistry, Polish Academy of Sciences, ul. Niezapominajek 8, 30239 Cracow, Poland; patrycja.wojton@ikifp.edu.pl (P.O.); pawel.wolski@ikifp.edu.pl (P.W.)

**Keywords:** multi-stranded DNA structures, stability, ligands, G-quadruplex, i-motif, molecular dynamics

## Abstract

This article provides a comprehensive examination of non-canonical DNA structures, particularly focusing on G-quadruplexes (G4s) and i-motifs. G-quadruplexes, four-stranded structures formed by guanine-rich sequences, are stabilized by Hoogsteen hydrogen bonds and monovalent cations like potassium. These structures exhibit diverse topologies and are implicated in critical genomic regions such as telomeres and promoter regions of oncogenes, playing significant roles in gene expression regulation, genome stability, and cellular aging. I-motifs, formed by cytosine-rich sequences under acidic conditions and stabilized by hemiprotonated cytosine–cytosine (C:C+) base pairs, also contribute to gene regulation despite being less prevalent than G4s. This review highlights the factors influencing the stability and dynamics of these structures, including sequence composition, ionic conditions, and environmental pH. Molecular dynamics simulations and high-resolution structural techniques have been pivotal in advancing our understanding of their folding and unfolding mechanisms. Additionally, the article discusses the therapeutic potential of small molecules designed to selectively bind and stabilize G4s and i-motifs, with promising implications for cancer treatment. Furthermore, the structural properties of these DNA forms are explored for applications in nanotechnology and molecular devices. Despite significant progress, challenges remain in observing these structures in vivo and fully elucidating their biological functions. The review underscores the importance of continued research to uncover new insights into the genomic roles of G4s and i-motifs and their potential applications in medicine and technology. This ongoing research promises exciting developments in both basic science and applied fields, emphasizing the relevance and future prospects of these intriguing DNA structures.

## 1. Introduction

The fundamental building blocks of organisms are proteins, composed of twenty different amino acids. Proteins are essential for the functioning of all cellular processes, serving structural roles, participating in nerve impulse transmission, and storing and transporting micro- and macroelements. The information needed to synthesize various proteins is contained in DNA. DNA, or deoxyribonucleic acid, is an important polymer that serves as the carrier of genetic information and is located in the nucleus of every living organism. DNA resembles a thread, composed of a large number of deoxyribonucleotides, each consisting of deoxyribose—a five-carbon sugar to which a phosphate group and one of four nitrogenous bases are attached: adenine (A), thymine (T), cytosine (C), or guanine (G). The bases act as carriers of genetic information, while the sugar and phosphate residues serve structural roles [1]. Since 1953, thanks to J. Watson and F. Crick, who determined the structure of DNA based on X-ray crystallography images, we have known the specific double helix. This is a fragment of DNA consisting of two polynucleotide chains that run antiparallel to each other, twisting around a common axis to form the double helix of Watson and Crick. The nitrogenous bases, oriented towards the interior of the helix, pair complementarily, meaning adenine pairs with thymine and cytosine always pairs with guanine. This complementary pairing ensures that the nucleotide sequence of one DNA strand determines the sequence of nucleotides in the other strand, which then twist helically around each other [2]. The dominant and most stable in vivo structure is the right-handed B-DNA, but under certain conditions, double-stranded DNA can adopt other double-helical forms, both right-handed and left-handed, such as A-DNA or Z-DNA [3].

Chemically and structurally similar to DNA, RNA (Ribonucleic Acid) plays crucial roles in various biological processes. However, RNA differs from DNA in a few key ways: RNA is typically single-stranded, unlike DNA. RNA contains ribose sugar, while DNA contains deoxyribose sugar. Additionally, RNA contains uracil instead of thymine, one of the four nitrogenous bases found in DNA. There are several types of RNA, each with their own specific function, including Messenger RNA (mRNA), Transfer RNA (tRNA), Ribosomal RNA (rRNA), MicroRNA (miRNA), Small Interfering RNA (siRNA), and Long Non-coding RNA (lncRNA) [4]. Due to space constraints, a more in-depth discussion of RNA will not be included in this review.

In the cell nucleus, DNA is packaged into chromosomes. A chromosome begins and ends with a segment composed of repeatedly occurring sequences of nucleic acids, known as telomeres [5]. These repeats are characterized by asymmetry in guanine and cytosine. In many telomeres, there are two to five repeats of guanine on one strand, paired with corresponding cytosines on the other strand. In humans, the telomeric repeat sequence is (TTAGGG):(CCCTAA). This allows us to distinguish between guanine-rich and cytosine-rich strands in the double-stranded telomeric segment of DNA. The guanine-rich strand is longer and forms a 3′ overhang at the end of the telomere [6]. The biological role of the telomere is to protect the chromosome from degradation or fusion with neighboring chromosomes, as well as to regulate cellular aging. Considering the results of studies, telomeres can also be viewed as prognostic markers for the process of tumorigenesis occurring at a very early stage of carcinogenesis [7]. Telomeres have a unique mode of replication, based on an enzyme called telomerase. Unlike cancer cells, which often express high levels of telomerase, this activity is strictly regulated in normal human somatic cells. Naturally, most telomeric DNA is double-stranded, except for the terminal part, where the 3′ overhang of the guanine-rich strand is single-stranded and forms a so-called T-loop [8]. With respect to these DNA fragments, under in vitro and in vivo conditions, depending on the sequence of the strands and environmental properties such as ionic strength of the solution, pH, and the presence of metal ions or other small organic molecules, we can observe the existence of other, less understood non-canonical higher-order DNA forms known as G-quadruplexes and i-motifs. The biological role of these structures is an area of active research. The aim of the aforementioned studies is to determine which alternative DNA conformations, including multi-stranded forms like triplexes or quadruplexes, exist in vivo. It is also important to determine how their formation is regulated and what information they convey [9].

Telomeric regions are one of the most well-documented areas where G-quadruplexes and i-motifs are found. Promoter regions of various oncogenes are rich in sequences capable of forming G-quadruplexes. Notable examples include the promoters of *c-MYC*, *KRAS*, and *BCL-2* genes [10,11,12]. These regions are crucial for the regulation of gene expression, and the formation of G-quadruplex structures can inhibit or promote transcription, depending on the context. This regulatory mechanism is a potential target for cancer therapeutics, as stabilizing G-quadruplexes in oncogene promoters can downregulate their expression. Similarly, i-motifs are present in the promoters of several oncogenes [13]. The formation of i-motif structures can also influence gene expression by acting as regulatory elements [14]. The dynamic nature of these structures allows them to act as molecular switches, responding to changes in cellular pH and other environmental factors [11].

Regulatory regions, including enhancers and silencers, often contain sequences that can form G-quadruplexes and i-motifs [14,15]. These regions are critical for the fine-tuning of gene expression and can influence the binding of transcription factors and other regulatory proteins. CpG islands, which are regions with a high frequency of cytosine–guanine dinucleotides, are particularly rich in potential G-quadruplex and i-motif-forming sequences [16]. Switch regions in immunoglobulin genes are another hotspot for G-quadruplex formation. These regions are involved in class switch recombination, a process essential for antibody diversity. The presence of G-quadruplex structures in these regions suggests a role in facilitating or regulating the recombination process [17].

This work aims to systematize the current knowledge related to the occurrence of non-canonical DNA forms, with particular emphasis on four-stranded structures. The vast amount of literature data concerning the structure, formation, stability, biological significance, and applications of multi-stranded, non-canonical DNA forms makes it challenging to draw definitive conclusions on these matters. Therefore, this work gradually discusses increasingly complex non-canonical forms, starting from two-stranded forms, through three-stranded forms, and reaching four-stranded forms, whose significance in nanomedicine and nanotechnology appears to be rapidly increasing. In this work, we strive to discuss only the most well-established and verified information regarding the properties of non-canonical DNA forms so that not only specialists, but also non-specialists can understand them and, if desired, refer to the cited literature for further knowledge on the topic. The issues addressed in this work mainly pertain to the stability and formation mechanisms of DNA tetraplexes. Therefore, significant attention is given to computer simulations as a tool that allows tracking these phenomena at the molecular level. These systems have proven exceptionally challenging to describe at the molecular level due to the complexity of possible mechanisms resulting from a complicated potential energy landscape exhibiting numerous local minima separated by high-energy barriers.

## 2. Double-Helical DNA Structures

**Figure 1:**
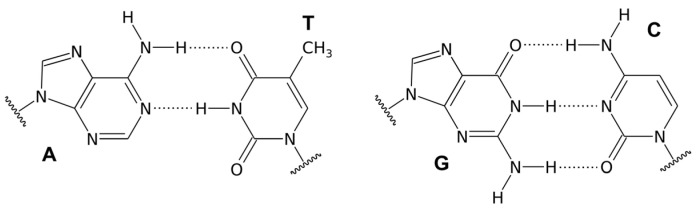
Watson–Crick hydrogen bonding interactions between complementary nitrogenous bases: A:T and G:C.

We distinguish three different forms of duplex deoxyribonucleic acid. The most widespread form, present in most DNA at neutral pH and physiological salt concentrations, is the right-handed double helix B-DNA model proposed by Watson and Crick. In this structure, two polynucleotide chains interact through Watson–Crick hydrogen bonds between the corresponding nitrogenous bases, forming complementary pairs. The bases fit the double helix model when a pyrimidine (C or T) on one strand is always paired with a purine (A or G) on the other strand. Adenine and guanine have a two-ring structure, while cytosine and thymine have only one ring. If adenine were to pair with guanine and cytosine with thymine, the distances between the two DNA strands would vary. However, when a single-ring molecule pairs with a double-ring molecule, the distance between the two strands remains constant, allowing the double helix to maintain a uniform shape. This specificity is characteristic of molecular recognition in the realm of nucleic acids. Adenine pairs with its complementary base, thymine through two Watson–Crick hydrogen bonds, while guanine pairs with its complementary cytosine through three hydrogen bonds (Figure 1). Double-stranded DNA structures are now well understood and extensively described in the literature [3].

**Figure 2:**
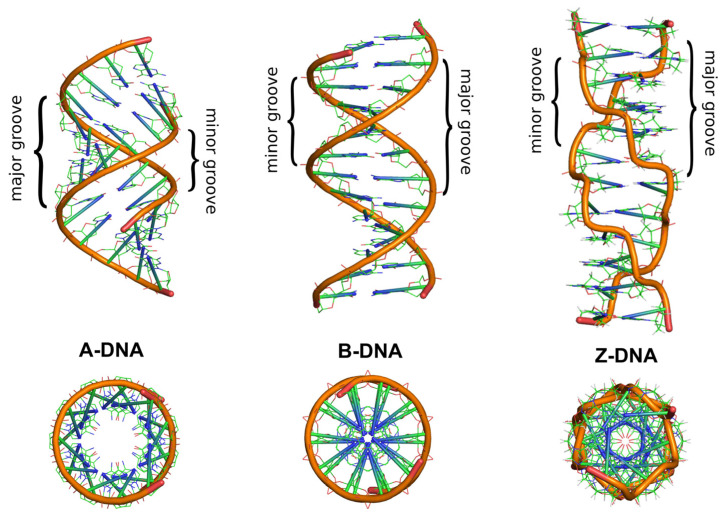
Models of the canonical forms of A-DNA, B-DNA, and Z-DNA using the sequence d (GC)_12_. View along the helix axis with distinct major and minor grooves, and top view showing the bases arranged in the center surrounded by the phosphate–sugar backbone.

In addition to the common right-handed B-DNA structure, the right-handed A-DNA form and the left-handed Z-DNA form are also known [18]. Figure 2 depicts these DNA structures and allows for tracing the similarities and key differences between them. In all these structures, the two polynucleotide chains helically wrap around a common axis. The chains are oriented antiparallel, in opposite directions. The 5′ end of one chain connects with the 3′ end of the complementary second DNA chain. In the double helix of B-DNA and A-DNA, pyrimidine and purine bases are located inside the helix, forming its core, while deoxyribose residues and phosphate groups are found on the outside. In the B-DNA structure, the planes of the sugar rings surround the bases and are arranged around the helix axis, whereas the planes of the bases are perpendicular to it. The B-DNA structure is the dominant type of structural conformation of DNA in cells [19]. A less common structural conformation is the A-DNA form, which DNA can adopt under dehydration conditions [1]. Although this helical structure is similar to B-DNA, it is much wider and flatter. The main difference between A-DNA and B-DNA structures is the different conformation of sugar residues. In the A-DNA structure, the C3′ carbon of deoxyribose is out of the plane of the furanose ring, adopting a C3′-endo (N-type) conformation, which consequently leads to the bases being tilted away from perpendicular to the molecular axis and different distances between adjacent phosphate residues.

Due to the tilt of the bases away from the perpendicular alignment to the helix axis, the A-DNA structure has an empty central core. Another difference between these DNA structures is the size of the minor groove in the helix loop. In B-DNA, it is narrow and deep, while in A-DNA helix, there is an almost complete disappearance of the minor groove, which is wide but shallow. This is due to fewer water molecules being bound by the phosphate groups compared to the B-DNA helix [18].

One of the significant questions in the field of molecular biology is whether multiple helical forms of DNA can coexist in vivo. Research described in the literature provides information on the possibility of interconversion between A-DNA and B-DNA under in vitro conditions. Increasing the concentration of organic or inorganic salt or decreasing the number of water molecules around B-DNA can lead to the transition of B-DNA to A-DNA in vivo [18]. In the late 1970s, the existence of another left-handed double-stranded DNA structure, called Z-DNA, was discovered in vitro. This is a radically different duplex structure, with two strands twisted into a left-handed helix and a distinct zigzag pattern (hence the name: Z-DNA) in the backbone. The appearance of the Z-DNA structure requires a sufficiently high concentration of organic and/or inorganic salt, the presence of appropriate concentrations of divalent ions (Mg^2+^, Zn^2+^), or monovalent ions (Na^+^), which help minimize the repulsive electrostatic interactions between the phosphates in the helix backbone. A necessary condition and characteristic feature for the formation of this DNA form is the presence of alternating, repetitive purine–pyrimidine dinucleotide sequences, such as GC and CG, which is different from other DNA structures. The repetition of such dinucleotide tracts enforces left-handedness and causes conformational differences in the sugar rings depending on whether they are connected to purines or pyrimidines [20,21]. In A- and B-DNA structures, each base pair is similarly oriented in the helix relative to its neighboring base pairs, making all pairs structurally similar. In Z-DNA, CG and GC dinucleotides have different twist angles, causing differences in base positioning in the double helix. In A- and B-DNA, the hydrogen-bonded bases are located inside the double helix, with the sugar rings and phosphate residues outside. Because all base pairs are similarly oriented relative to other base pairs in the same helix, A- and B-DNA have a repeating unit consisting of one nucleotide. In Z-DNA, the repeating unit is two base pairs. The CpG dinucleotide differs from the GpC dinucleotide. In Z-DNA, there are 12 base pairs per helical turn, which should give an average helix twist of −30°, where the negative value indicates a left-handed rotation in Z-DNA compared to the positive rotation found in right-handed helices. However, the twist angle for CpG is only −9°, while the twist angle for GpC is −51°. Consequently, each base pair in Z-DNA is not similarly oriented in the helix relative to neighboring base pairs. However, the two base pairs are each oriented in the same way as the next two adjacent base pairs. The twist angle for two base pairs relative to the next two is −60°. The orderly base arrangement in the helix center seen in right-handed DNA structures does not exist in Z-DNA. Analysis of the CpG dinucleotide arrangement revealed that cytosines are oriented towards the helix center, while guanines are positioned above and below the deoxyribose rings. In the GpC dinucleotide, the bases are relatively similarly arranged. Thus, base positioning is a distinguishing feature of Z-DNA in right-handed structures. In A- and B-DNA, bases are protected from the solvent by being located inside the helix. In Z-DNA, some ring positions are much more exposed to the solvent and interaction with other biomolecules due to their distinctive positioning compared to A- and B-DNA. The major and minor grooves in Z-DNA, unlike in A or B-DNA, show little difference in widths [22]. The Z-DNA conformation is quite challenging to analyze because it does not exist in vivo as a stable feature of the double helix. Instead, it is a transient structure, sometimes induced by biological activity, and then quickly disappears [23]. The biological significance of Z-DNA has been controversial since its discovery, with many early studies raising as many questions as they answer [24]. However, available literature indicates that DNA structures other than B- or A-DNA have significant (still being discovered) roles in biochemical processes [25,26,27].

## 3. Triple-Helical DNA Structures

DNA strands are dynamic structures that undergo various topological transformations in vivo. During biological processes occurring in the cell nucleus, the double-stranded structure of DNA undergoes changes, including replication, unwinding, and then reformation of the double helix. During this time, local changes occur in the DNA strands, which may later prove to be significant. These changes are related, among others, to reversible twisting, bending, and stretching of the helix. As a result of the interaction between strands through hydrogen bonding, forms of DNA other than those described above can form. These forms can involve fragments of the same DNA with specific sequences or new, additional strands of nucleic acids. In this way, triple-stranded structures, called triplexes, and four-stranded structures, called quadruplexes, can form [28,29,30].

The formation of triple-helical DNA structures can be associated with conformational changes occurring in the double helix, involving the ability of a third DNA strand to associate in the major groove of the duplex structure. Triplexes are thus primarily composed of two antiparallel strands, connected by Watson–Crick hydrogen bonds as in B-DNA. The difference compared to the common double helix is the deviation from the axis and some unwinding of the helix caused by the spatial interference associated with the association of the third DNA strand.

**Figure 3:**
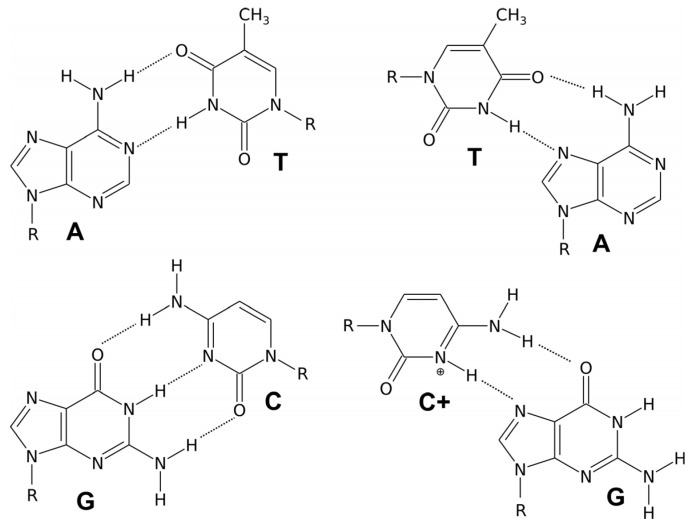
Watson–Crick and Hoogsteen base pairs. On the left are the traditional Watson–Crick AT and GC base pairs. On the right are the Hoogsteen TA and C+G base pairs. For cytosine to pair with guanine in a Hoogsteen bond, the N3 position of cytosine in the third strand must be protonated. The base pairing sites involved in Hoogsteen bonds in a purine molecule differ from those involved in Watson–Crick pairing (Hoogsteen pairing involves the N7 position in the imidazole ring) [22].

Triple-stranded nucleic acid structures were discovered in 1956 by Davies and Rich. For the formation of a triplex, a necessary condition is the presence of double-stranded complementary polypurine–polypyrimidine tracts. The third strand appearing in these structures can be oriented relative to the duplex strand it interacts with in a parallel manner, meaning the 3′ end of the third strand interacts with the 3′ end of the duplex DNA fragment by forming Hoogsteen hydrogen bonds, seen in Figure 3, or in an antiparallel manner, where the 3′ end of the third strand forms reverse Hoogsteen hydrogen bonds with the 5′ end of the DNA fragment [31].

Complementary interactions related to the formation of hydrogen bonds are responsible for the specific connection of the duplex with the third DNA strand. Since Watson–Crick base pairing sites are already engaged in hydrogen bonds in the double helix, the third strand must bind through Hoogsteen base pairing with other sites in the duplex. The middle strand of the triplex must be rich in purine because pyrimidine does not have two surfaces capable of forming more than one hydrogen bond. Therefore, triple DNA requires a homopurine–homopyrimidine DNA region. If the third strand is rich in purine, it forms reverse Hoogsteen hydrogen bonds in an antiparallel orientation with the purine strand of the Watson–Crick helix. When the third strand is rich in pyrimidines, it forms Hoogsteen bonds in a parallel arrangement with the purine strand paired with the Watson–Crick duplex [22].

Formation of a Hoogsteen hydrogen bond (Figure 3) with cytosine already engaged in a Watson–Crick hydrogen bond requires the protonation of the N3 nitrogen of cytosine in the third strand, which only occurs in an acidic environment with a pH below 4.5 [22,32]. Karst Hoogsteen discovered the formation of these types of hydrogen bonds after observing the crystal structure of a nucleic acid complex in which adenine and thymine analogs formed base pair motifs with different geometry than the Watson–Crick motif. Hoogsteen base pairs have significantly different geometry than Watson–Crick base pairs, leading to different behavior. Hoogsteen pairs exist transiently in some forms of DNA in thermal equilibrium with standard Watson–Crick base pairs. They are also observed in protein–DNA complexes and G-quadruplex structures [22,33].

DNA triplexes can be categorized based on the origin of the third strand. If the third strand originates from an independent molecule, known as the triplex-forming oligonucleotide (TFOs, triplex-forming oligonucleotides), and binds to a double-stranded DNA (dsDNA), it forms an intermolecular triplex. In contrast, when the third strand is part of a single strand that also contains the dsDNA, the structure is called an intramolecular triplex. Intramolecular triplex DNA, H-DNA, is formed from a duplex with homopurine and homopyrimidine strands with mirror repeat symmetry. In DNA, hydrogen bonds between the two helices are typically Watson–Crick bonds, while the bonds between the duplex and the TFO are either Hoogsteen or reverse-Hoogsteen bonds. As mentioned, the TFO can bind to the DNA strand in either a parallel or antiparallel orientation, depending on its directionality relative to the strand forming hydrogen bonds [32].

For the stabilization of triple-helical DNA structures, the presence of metal ions such as Ca^2+^, Mg^2+^, and Zn^2+^ and polyamines is required, which are responsible for neutralizing the negative charges of the phosphate groups. Complete base complementarity in all three chains is also required, as even a single mismatch can cause significant destabilization of the structure. Purine-rich triplexes usually involve purine bases (adenine and guanine) in the Watson–Crick double-stranded helix and in the third strand. The third strand binds through Hoogsteen or reverse Hoogsteen interactions. Guanine–cytosine (G·C) and adenine–thymine (A·T) base triplets are commonly formed. Due to the stronger hydrogen bonds in G·C base triplets and more effective base stacking interactions, purine triplexes tend to have higher thermal stability and melting temperatures (*T_m_*). The thermodynamic favorability of these interactions makes purine triplexes generally more stable. Pyrimidine-rich triplexes often have cytosine and thymine in the third strand. Pyrimidine triplexes generally involve cytosine in the third strand, forming Hoogsteen bonds with guanine in the double helix, and thymine interacting with adenine. Pyrimidine triplexes are thermodynamically less stable due to weaker A·T triplet bonds and the need for cytosine protonation. However, under optimal pH conditions (acidic environments), pyrimidine triplexes can become more stable. In DNA triplexes, the sugar conformation (the shape of the deoxyribose ring) influences helix stability. Sugars adopting the C3′-*endo* conformation (typical for A-DNA) stabilize triplexes, while C2′-*endo* is less stabilizing, favoring the B-DNA form of the duplex [32]. Moreover, purine–pyrimidine inversion poses a significant challenge in the design and stabilization of DNA triple helices, particularly in the context of triplex-forming oligonucleotides (TFOs). A purine–pyrimidine inversion in the duplex, i.e., changing a purine pair to a pyrimidine pair, positions the purine outside the hydrogen bonding range of the third strand base. As a result, the triplex structure becomes unstable and unsuitable for bioactivity. Numerous attempts have been made to design base analogs to overcome this limitation [34,35,36].

**Figure 4:**
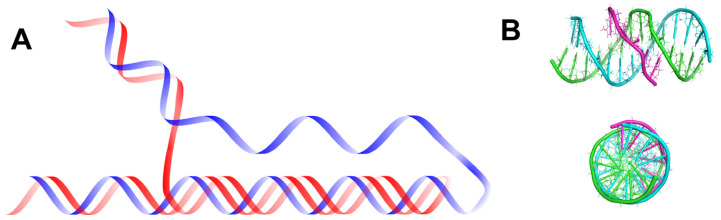
(**A**) H-DNA, intramolecular triple-stranded DNA. Within the polypurine–polypyrimidine helix with mirror symmetry of repeats, one of the single strands (marked in red) bends and forms a triple structure, while the other strand (blue) remains unpaired [37]. (**B**) Molecular conformation of a parallel DNA triple helix with 5′ and 3′ triplex–duplex junctions [38].

Naturally occurring intramolecular triplex structures, referred to as H-DNA, form when part of the mirror sequence dissociates into single, separate pyrimidine and purine strands (Figure 4), followed by the reassociation of the homopyrimidine strand with the remaining mixed duplex part, leading to the formation of a parallel triplex structure in the major groove. The other half of the homopurine strand remains single-stranded. There are also cases where the homopurine strand re-associates, forming *H-DNA, i.e., an antiparallel triplex [37]. In the 1980s, Frank-Kamenetskii and colleagues demonstrated that in addition to required base complementarity, the homopurine–homopyrimidine region in H-DNA must contain mirror repeat symmetry. Mirror repeats occur when a DNA fragment of one strand has the same base sequence reading in both 3′ and 5′ directions starting from a defined central point for that segment (the sequence GAA AAG represents mirror repeats).

Triple-stranded structures are described in many review papers and monographs. H-DNA plays an important in vivo role and is inherently mutagenic and recombinogenic, making elements of H-DNA potentially pharmacologically exploitable. The occurrence of H-DNA has been confirmed, among other instances, during studies of the hsp26 promoter gene in fruit flies. These studies identified the importance of the purine–pyrimidine tract for proper gene activation and chromatin formation [39,40]. The formation of triple-stranded H-DNA structures in vivo does not require the presence of specific proteins but depends on sufficiently long purine–pyrimidine tracts. In eukaryotic organisms, DNA exists in a supercoiled form tightly packed in nucleosomes, where it is associated with histone proteins that hinder the formation of naturally occurring stable higher-order structures. Triple-stranded structures can also be induced artificially by introducing complementary oligonucleotides into cells (TFOs). Oligonucleotides forming triple helices bind to specific sequences in double-stranded DNA via hydrogen bond interactions. It has been shown that TFOs reduce gene expression, induce targeted modifications of genomic DNA, and stimulate DNA recombination. Additionally, they can be used as carriers to position DNA-modifying agents at selected sequences. Effects mediated by TFOs have primarily been described in cell culture, although one study demonstrated TFO activity in a mouse model. It was found that TFOs introduced into adult mouse somatic cells can induce genome mutations in specific regions, confirming that modifications of the genome induced by the formation of triple-stranded structures can occur in animal organisms. This finding was a significant step towards the development of gene therapy and the use of triplex formation in medical applications [41]. Critical issues concerning TFO-based technologies include the development of new analogs of oligonucleotides with improved binding affinity, better target selectivity, and sufficient stability in the intracellular environment. The enforced formation of triple-stranded structures such as TFOs has found application in biochemistry and molecular biology. It has been demonstrated, for example, that the creation of triplex structures in promoter regions blocks access to transcription factors and inhibits gene activation in vitro. Introducing complementary oligonucleotides, forming triplex structures (TFOs), into cells allows for the correction and silencing of genes by inducing lasting changes in their sequences [39]. The natural formation of intramolecular triple-stranded helical DNA structures as well as their induction through complementary oligonucleotides remains an intriguing research topic due to their fascinating biological functions and pharmacological potential in gene therapy, as well as their ability to engineer functional DNA-based nanomaterials with precision [42]. Various studies have employed triplex formation to inhibit gene expression [43], detect sites of DNA damage due to mismatches in single bases [44], and inhibit protein–DNA binding [45]. The biologically functional form of DNA triplexes is a three-stranded complex, which acts as an intermediate in DNA recombination processes. These complexes are stabilized by the RecA protein [46], which facilitates the pairing of a single-stranded DNA molecule with a homologous region on double-stranded DNA, forming a stable triplex structure necessary for genetic recombination and DNA repair. They also have been implicated in other cellular processes, such as DNA replication and transcription [47]. Triplex structures are also used in the development of DNA nanotechnology areas and functional materials based on nucleic acids. Triple structures of nucleic acids are important elements in designing materials responsive to stimuli. Integrating triple-stranded DNA structures with acrylamide polymers has led to the synthesis of pH-responsive hydrogels that undergo reversible gel–sol transitions and exhibit shape memory properties. These new materials hold great promise as carriers for controlled drug delivery or for encoding information and creating intelligent materials for engineering applications [48,49]. Research has also been conducted using functionalizing dendrimer nanoparticles with triple-stranded nucleic acids, which actively participate in oncogene silencing and tumor reduction in a mouse model of cancer. These studies underscore the importance of DNA triplexes in future nanomedicine applications [43,50].

## 4. Four-Stranded DNA Structures: G-Quadruplexes and i-Motifs

A eukaryotic chromosome, which is a compact form of DNA wound around histone proteins and located in the cell nucleus, begins and ends with a specific nucleoprotein structure called a telomere. Telomeres protect chromosome ends from degradation or fusion with neighboring chromosomes during cell divisions and regeneration processes and are responsible for genome integrity. Telomere length shortens with age, which is associated with so-called replicative aging—the gradual loss of a cell’s ability to divide. Progressive telomere shortening leads to cell aging or apoptosis. Shorter telomeres can also affect genome instability and oncogenesis. The rate of telomere shortening is crucial for health, the aging rate of an individual, and potentially responsible for cancer. Telomeres have a unique replication mode based on an enzyme called telomerase. Telomerase activity decreases with cell aging. Elevated telomerase activity is observed in cancer cells, allowing an unlimited number of cell divisions, which can result in tumor formation [51,52]. The telomeric DNA segment is characterized by repetitive sequences of nucleotides (TTAGGG):(CCCTAA) forming a double-stranded DNA helix. Most of the telomeric DNA segment is double-stranded except for the terminal part, where the 3′ strand rich in guanine is longer and single-stranded. Telomeric sequences are associated with a series of proteins that determine their proper shape and stability. The presence of the protective protein complex causes internal bonds to form in the telomere, leading to the formation of two loops, T (T-loop) and D (D-loop), which are responsible for telomere structure stabilization. The double-stranded telomeric part, due to the activity of the protective protein complex, loops and closes into a larger T-loop. Meanwhile, the free 3′ end strand within the T-loop binds to the double-stranded telomeric DNA segment, forming a smaller D-loop [53,54]. Telomeric DNA segments are characterized by guanine and cytosine asymmetry. In the nucleotide sequence repeats in telomeres, there may be from two to five adjacent guanine repeats on the same DNA strand, with corresponding cytosines on the opposite strand [9,51,55]. Telomeric strands rich in guanine and cytosine can form higher-order DNA structures. The guanine-rich strand can adopt a four-stranded G-quadruplex structure, while the cytosine-rich strand can form a so-called i-motif [56,57].

The discovery of G-quadruplexes dates back to the early 1960s when studies demonstrated that guanine-rich DNA sequences could form stable secondary structures. The ability of guanine-rich sequences to self-aggregate into a four-stranded structure was first noted in fiber diffraction studies, which revealed that guanylic acid derivatives could form right-handed helical structures [58]. These structures were later shown to be stabilized by Hoogsteen hydrogen bonding between guanine bases, leading to the formation of G-tetrads [56]. The in vivo discovery of G-quadruplexes occurred much later. The development of structure-specific antibodies, such as the BG4 antibody, allowed researchers to visualize G-quadruplex structures directly in living cells. In 2013, G-quadruplexes were first detected in the nuclei of human cells using immunofluorescence techniques with the BG4 antibody, which selectively binds G-quadruplexes. This provided the first direct evidence of G-quadruplex structures forming dynamically in living cells, particularly at telomeres and promoter regions of highly transcribed genes [59]. I-motifs, a counterpart to G-quadruplexes, were first described in vitro using NMR spectroscopy in the early 1990s [60]. The in vivo discovery of i-motifs occurred only recently. In 2018, using an i-motif-specific antibody, researchers were able to visualize i-motif structures in the nuclei of living human cells [61].

The G-quadruplex is a non-canonical secondary structure of DNA, biologically significant for DNA replication, transcription, and telomere stability, making it a potential target in new disease treatment methods. This structure forms spontaneously through the interaction of sequences containing continuous guanine repeats and is characterized by the presence of two or more stacks of four guanines, forming a planar quartet [62]. The G-quadruplex is stabilized by Hoogsteen hydrogen bonds and additionally by a monovalent ion of appropriate size, such as Na^+^ or K^+^, located in the central core of the guanine quartet, neutralizing the electrostatic repulsion of the negative oxygen charge in guanine [56,63,64]. Crystallographic studies have been valuable in explaining the role and location of monovalent metal ions in stabilizing G-quadruplex structures. Feigon [65] and colleagues, analyzing the sequence d(TGGGGT), found that K^+^ ions positioned between the planes formed by guanine quartets bind to them through eight coordination bonds, stabilizing this structure. Crystallographic studies also demonstrated that the human telomeric sequence (TTAGGG) forms intramolecular G-quadruplexes in solutions in the presence of Na^+^ ions at near-physiological concentrations [66]. Although G-quadruplexes were first discovered in vitro, increasing evidence suggests that this unique nucleic acid structure is also formed in living cells. Gavathiotis and colleagues confirmed the formation of G-quadruplex structures in human nucleotide sequences found in telomeric parts of DNA through NMR studies and molecular dynamics simulations [67]. Several research groups have conducted computational analyses to find potential non-telomeric G-quadruplex-forming sequences in the genome. Huppert and Balasubramanian discovered that there are over 300,000 potential G-quadruplex-forming sequences in the human genome. These sequences are not randomly located; they are found in functional genomic regions such as promoters, introns, and untranslated regions of genes [68,69].

**Figure 5:**
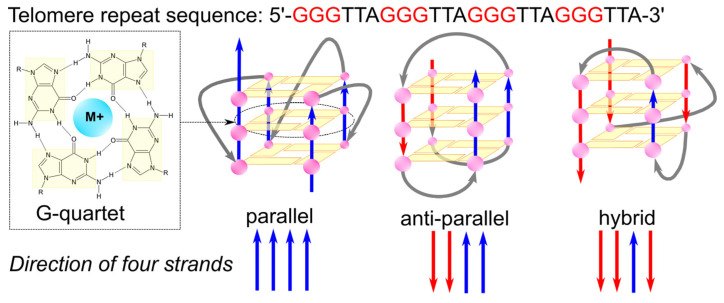
Schematic structures of G-quadruplexes. These structures are represented by three stacks of guanine quartets and are categorized into three types: parallel, antiparallel, and hybrid, according to strand orientation. In the quartet shown on the left, the symbol M+ indicates the position of a monovalent metal ion, and the dotted lines represent Hoogsteen hydrogen bonds between guanines [71]. (Adapted with permission from Ref. [71] Copyright 2020 Elsevier).

G-quadruplex structures are diverse. The conformation of a G-quadruplex is determined by the orientation of the glycosidic bonds of guanines in the quartets, with the parallel, antiparallel, or mixed orientation of the strands contributing guanine bases to the quartets, and the length and sequence of the loops connecting the series of guanines, as shown in Figure 5. G-quadruplexes exhibit significant thermodynamic stability, largely derived from the stacking of hydrophobic quartets and modulated by the length and sequence of the loops [62,70].

The glycosidic torsion angles, specifically syn and anti conformations, are crucial in G-quadruplex formation as they influence G-tetrad arrangement and strand polarity. In parallel G-quadruplexes, all G residues in a tetrad adopt the same glycosidic angle (typically anti) to form a stable planar structure held by Hoogsteen hydrogen bonds. However, antiparallel G-quadruplexes require a mix of syn and anti conformations for G-tetrad formation. This interplay between strand polarity and torsion angles helps direct G-quadruplex folding, often through the strategic incorporation of G analogs that favor either syn or anti conformations [72]. Modification of the base can have a significant impact on the structure, stability, and function of G-quadruplexes. For example, the insertion of bulky substituents (atoms or groups) to the C8 position of guanine strongly favors its syn conformation around the glycosidic bond [73]. This shift can either stabilize or destabilize the G-quadruplex, depending on the specific sequence. Xu et al. [74] showed that substitutions of 8-methylguanine at positions that exist in syn conformations in antiparallel G-quartets stabilize the structure in the G-rich strand. On the other hand, Dias et al. [75] found that if these 8-substituted analogs replace an anti-G of the quadruples, it will destabilize the quadruplex.

Another critical factor affecting the stability and folding topologies of G-quadruplexes is loop length. Risitano et al. discovered that G-quadruplex sequences with longer loops tended to form more stable structures and exhibited faster folding kinetics, with minimal hysteresis during melting and annealing [76]. Generally, even slight modifications in loop length and sequence can result in significant differences in quadruplex stability and folding dynamics.

The formation of G-quadruplexes occurs in single-stranded regions, where they arise during replication and transcription as a result of folding. The resulting structure, characterized by a large diameter and four grooves, defines the uniqueness of the G-quadruplex, distinguishing it from duplex DNA. The formation of a G-quadruplex in the 3′ overhang of the human telomere can contribute to inhibiting the overexpression of telomerase, which is characteristic of cancer cells. Therefore, the stabilization and promotion of this non-canonical form of DNA present a promising strategy for developing new cancer therapies [77,78,79]. Due to this potential, the topic of G-quadruplexes is widely discussed in the literature, with researchers focusing on understanding the structural topology of G-quadruplexes, factors that promote their stabilization, and the therapeutic and diagnostic potential of this unique nucleic acid structure [80].

G-quadruplexes are involved in regulating a variety of biological functions, including telomere maintenance, DNA replication, transcription, recombination, epigenetic regulation, etc. [69,81]. They interact with cellular proteins such as helicases, transcription factors, and epigenetic modifiers to control these processes. G-quadruplex formation in cells is dynamic and dependent on cell type and state. Their biological roles are mediated by a variety of cellular proteins that specifically recognize, bind, stabilize, or unwind G-quadruplex structures [69,81].

A variety of helicases play essential roles in resolving G-quadruplex structures, which is crucial for DNA replication, transcription, and genome stability. For example, WRN, BLM, and DHX36 (RHAU) are well-studied helicases that bind to and unwind G-quadruplexes. These enzymes have evolved mechanisms to specifically recognize and unfold G-quadruplex structures. For instance, the helicase DHX36 uses a unique DHX36-specific motif (DSM) to bind G-quadruplexes, particularly at the *MYC* promoter. The unwinding of G-quadruplexes by these helicases prevents genomic instability and DNA damage, which may otherwise arise if G-quadruplex structures are left unresolved during processes such as DNA replication [69,81].

Telomeric G-quadruplexes are particularly enriched at chromosome ends, where they play a role in telomere maintenance. Proteins like POT1, RPA, and the CST complex are key players in managing telomeric G-quadruplex structures. POT1, for example, unfolds G-quadruplex structures at the telomere, allowing telomerase access for telomere extension, thus preventing telomere shortening and genome instability [69,81].

Several transcription factors and coactivators bind to G-quadruplex structures in gene promoters, influencing gene expression. Proteins like nucleolin stabilize the G-quadruplex in the *MYC* promoter, leading to transcriptional repression [81], while others like hnRNP A1 and MAZ can bind and resolve G-quadruplexes at the *KRAS* promoter. This interaction controls the transcriptional activation of key oncogenes and other regulatory genes, often making G-quadruplex structures essential nodes in transcriptional regulation [69].

G-quadruplexes also influence epigenetic regulation by interacting with chromatin, remodeling proteins and enzymes like ATR-X and DNA methyltransferases (DNMTs) [69]. ATR-X binds to G-quadruplex-rich regions, such as CpG islands and tandem repeats, helping resolve G-quadruplex-induced replication stress. DNMT1, in particular, binds to G-quadruplex structures and loses its enzymatic activity, thus altering DNA methylation patterns and potentially regulating gene expression through epigenetic modifications [81].

Scientists are also researching aptamers, which, when constructed from guanine-rich DNA fragments, can form stable G-quadruplex structures under physiological conditions and recognize various proteins [82]. Aptamers are small oligonucleotides based on DNA or RNA, typically produced by Systematic Evolution of Ligands by Exponential Enrichment (SELEX). This method involves obtaining oligonucleotides with high affinity for a target sequence, initially generating a preliminary panel of aptamers, and then conducting next-generation sequencing over several cycles to obtain a range of potentially useful aptamers. The term aptamer comes from the Latin word “aptus”, meaning “to fit”, and the Greek word “meros”, meaning “part”. The structures of aptamers provide specific binding sites for small molecules or macromolecular compounds of various types, including cells, cell surface proteins, bacteria, and viruses. Additionally, aptamers interact with targets with high affinity and selectivity [83].

Over the past few years, a variety of aptamers forming G-quadruplex structures have been developed, whose potential has been harnessed in numerous ways, including as anticoagulants, as therapeutic agents for treating diseases, and as nanodevices. Studies involving aptamers have shown that aptamers forming G-quadruplexes are strong and useful alternatives to antibodies in targeted therapy, as well as in in vitro and in vivo diagnostics or biomarker detection. Their high stability, increased cellular uptake, ease of chemical modification, low production costs, and convenient storage contribute to this. One example of a G-quadruplex-based aptamer is AS1411, formally known as ACT-GRO-777 [84]. Discovered accidentally rather than through the conventional SELEX approach, AS1411 is the most advanced and first-in-class anticancer aptamer to enter clinical trials. AS1411 is a G-quadruplex-forming 26-mer DNA aptamer, highly stable and resistant to nuclease degradation (an enzyme that cleaves phosphodiester bonds in nucleic acids), targeting nucleolin. Nucleolin is a ubiquitous and multifunctional protein that plays a crucial role in cell survival, growth, and proliferation [85]. AS1411 binds to the external domain of nucleolin, which is overexpressed on the surface of cancer cells. Therefore, this aptamer can specifically recognize and then be internalized by cancer cells [86]. The internalized aptamer–nucleolin complex inhibits DNA replication, causing cell accumulation in the S phase and cytotoxicity towards cancer cells [87]. Researchers have also proposed another mechanism of action for this aptamer: AS1411 is internalized by nucleolin-mediated macropinocytosis. The internalized aptamer–nucleolin complex causes hyperstimulation of macropinocytosis, which then induces non-apoptotic cell death known as methuosis [88]. In preclinical studies, AS1411 demonstrated antiproliferative effects in several cell lines, including lung, prostate, and breast cancer cells. In phase I clinical trials, AS1411 was well tolerated by patients with advanced solid tumors without severe toxicity [84]. In phase II clinical trials, AS1411 showed promising activity against metastatic renal cell carcinoma and acute myeloid leukemia with minimal toxicity [89].

Given that most anticancer and antiviral drugs are associated with severe side effects due to their poor selectivity, there is an urgent need for effective drug delivery systems. Aptamers, which have the ability to quickly recognize protein targets, could help achieve drugs with specific targeting. Aptamers can be conjugated with drugs to deliver them to specific receptors, which are typically surface proteins that are overexpressed on target cells and underexpressed on healthy, non-target cells [90]. An interesting case is the previously discussed aptamer AS1411, which acts both as a potential drug with promising anticancer and anti-HIV activity [91], and as a drug delivery system that is efficiently and selectively internalized by nucleolin, overexpressed on cancer cells, through macropinocytosis [92].

Although guanine-rich aptamers have a wide range of applications, the molecules still face certain challenges, such as nuclease degradation, renal clearance, and some hesitancy before transitioning to a new type of product [93]. Therefore, additional research is required, particularly at the preclinical and clinical levels, for guanine-rich aptamers to be widely used in the future, especially in therapeutic and diagnostic fields.

With the increase in research and knowledge, scientists have discovered that RNA can also fold into G-quadruplex structures. Gq-RNA shares key structural features with Gq-DNA; however, one observed difference is the higher thermodynamic and thermal stability of Gq-RNA compared to its DNA counterpart. These differences are thought to be due to better stacking of guanine tetrads and the presence of an additional network of hydrogen bonds involving the extra OH groups in RNA ribose. Additionally, guanine-rich regions in RNA form G-quadruplexes that are more compact and less hydrated, which has made these structures a recent research trend [94,95,96]. Research on the conditions favoring the formation of G-quadruplex structures in both DNA and RNA, as well as the ligands stabilizing them, is of great interest to scientists and a source of lively debate, as they are considered a real therapeutic strategy against cancer and neurological disorders [97].

Hybrid RNA–DNA G-quadruplexes, composed of both RNA and DNA strands, exhibit unique structural characteristics. RNA loops within these structures can introduce conformational flexibility and stability. The G-quartets themselves can be composed of either DNA or RNA nucleotides, potentially affecting stacking interactions and overall stability. Additionally, the groove dimensions of hybrid RNA–DNA G-quadruplexes may differ from those of DNA-only G-quadruplexes due to variations in the sugar–phosphate backbone and base pairing. Interestingly, bioinformatics studies have revealed the potential prevalence of intermolecular hybrid DNA–RNA G-quadruplexes in humans [98,99].

G-quadruplexes in eukaryotic species have been widely studied, but their presence in bacteria and viruses has only attracted attention in recent years [100,101,102,103]. In bacteria, G-quadruplexes are located in regulatory regions, playing important roles in replication, radiation resistance, antigenic variation, and latency [102]. In viruses, they play a regulatory role in key viral stages [104]. Recent studies have demonstrated the formation and function of G-quadruplexes in pathogens responsible for serious diseases, including Pseudomonas aeruginosa [105], human papillomavirus (HPV) [106], human immunodeficiency virus (HIV) [104], and SARS-CoV-2 [107].

The structure of guanine tetrads was identified in the early 1960s, and by the 1990s, G-quadruplexes were an intriguing, little-known deviation from the canonical Watson–Crick structure. However, over the past two decades, an extraordinary amount of information has been obtained, allowing the advancement of knowledge about this nucleic acid structure from basic construction to clinical applications.

Bioinformatics tools like QGRS Mapper [108], G4Hunter [109], QuadBase [110], and Pqsfinder [111] have been instrumental in identifying and characterizing G-quadruplexes in the human genome. These tools have facilitated the discovery of thousands of potential G-quadruplex-forming sequences, providing valuable insights into their structural features, biological functions, and potential therapeutic applications. By using these tools, researchers have been able to uncover the widespread presence of G-quadruplexes in various genomic regions, including promoters, coding sequences, and telomeres. Furthermore, these tools have helped researchers to explore the role of G-quadruplexes in gene regulation, telomere maintenance, and other biological processes.

Several sequencing methods have been developed to identify and characterize G-quadruplexes in the genome. These methods provide valuable insights into the distribution, structure, and function of G-quadruplexes. ChIP-seq (Chromatin Immunoprecipitation Sequencing) [112] is used to identify proteins that bind to specific DNA sequences, including Gq. Antibodies against proteins known to interact with Gq are used to pull down DNA fragments associated with these proteins. The DNA is then sequenced to determine the genomic locations of the bound proteins. ChIP-seq can be used to identify regions in the genome where proteins like the DDX5 helicase [113] bind, suggesting the presence of Gq. ICLIP (individual-nucleotide resolution cross-linking and immunoprecipitation) [114] is a high-resolution method used to map the binding sites of RNA-binding proteins on a transcriptome-wide scale. By using antibodies against RNA-binding proteins known to interact with G-qudaruplex, iCLIP can identify RNA molecules containing G-quadruplex and the precise nucleotides involved in the interactions [115]. SMRT sequencing (Single Molecule, Real-Time sequencing) [116] is a long-read sequencing technology that can directly detect DNA modifications, including those associated with G-quadruplex structures. SMRT sequencing can be used to detect G-quadruplexes based on changes in the polymerization rate or fluorescence signals during sequencing. These example methods, when combined with computational predictions and biochemical assays, provide a powerful toolkit for studying the role of Gq in various biological processes, including gene regulation, DNA replication, and telomere maintenance.

To visualize G-quadruplexes in living cells, researchers mainly use G-quadruplex-specific antibodies like BG4 [59], small-molecule fluorescent probes such as Thioflavin T [117], pyridostatin [118], or advanced microscopy techniques such as super-resolution microscopy and FRET [119]. These tools allow real-time observation of G-quadruplex dynamics and distribution within cells, providing valuable insights into their biological roles in processes like transcription, replication, and genome stability.

Despite this progress, research on G-quadruplexes still requires further investigation into their structure, biological function, and the identification of small-molecule ligands that could facilitate the formation and stabilization of G-quadruplex structures, as these are necessary for understanding and developing new treatment methods, such as cancer therapy, viral infection control, and targeted drug delivery.

**Figure 6:**
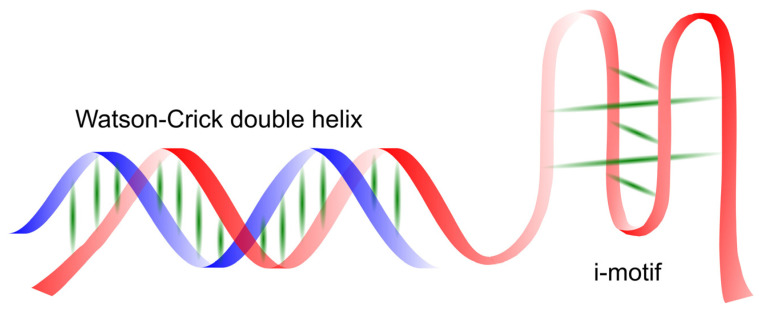
Schematic comparison of the Watson–Crick double helix with the i-motif structure [12]. (Adapted with permission from Ref. [12], Copyright 2014 Elsevier).

In genomic DNA, wherever a guanine-rich sequence is present, complementary cytosine-rich sequences are also found. These sequences can also form four-stranded structures known as i-motifs, as shown in Figure 6. Less is known about the occurrence of these structures in vivo and their potential as targets for chemical intervention in cell biology compared to G-quadruplex structures. The name i-motif (intercalated motif) refers to the phenomenon of intercalation, leading to characteristic DNA twisting and the pattern or motif describing how this occurs [12].

**Figure 7:**
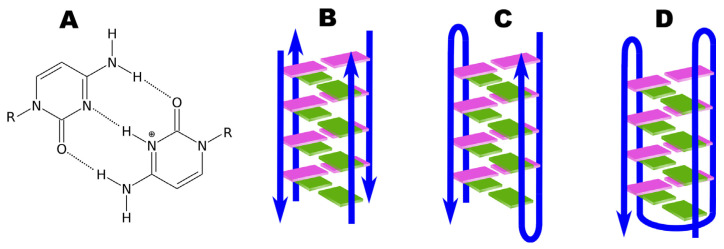
Schematic illustration of i-motif conformations: (**A**) hemi-protonated cytosine–cytosine+ base pair; (**B**) tetramolecular i-motif structure; (**C**) dimeric i-motif structure; (**D**) intramolecular i-motif structure [121]. (Adapted with permission from Ref. [121], Copyright 2014, American Chemical Society).

The first DNA i-motif was characterized by Gehring et al. for the hexamer sequence d(TCCCCC), forming an intercalated four-helical tetramolecular structure under acidic conditions [60]. The i-motif consists of two parallel DNA duplexes held together in an antiparallel orientation by the intercalation of protonated cytosine–cytosine (C-C+) base pairs. This structure can be formed by the spatial arrangement of C-C+ base pairs involving cytosine repeats present in a single nucleic acid strand, creating an intramolecular i-motif. On the other hand, an i-motif can also be formed by the interaction of cytosine repeats present in two (dimers) or four (tetramers) independent nucleic acid strands, forming intermolecular i-motif structures (see Figure 7) [120,121].

The intercalation of base pairs in two parallel duplexes leads to a structure with two main wide grooves and two smaller, narrow grooves. The two smaller grooves are very narrow, causing numerous short distances between the strands along the phosphate backbones. This results in destabilizing interactions due to the close proximity of adjacent negatively charged phosphate backbones that delineate the smaller groove. This must be balanced for the i-motif to be stable. Molecular dynamics simulations were used to investigate the impact of phosphate repulsion on the stability of the tetramolecular i-motif formed from the d(CCCC) sequence. These simulations showed that van der Waals forces and hydrogen bonds between the sugars are responsible for stabilizing the narrow grooves in the i-motif structure [122].

Hemi-protonated C-C+ base pairs are also important for the stability of the i-motif. The three hydrogen bonds of the C-C+ base pair confer high stability. Computer calculations indicate that the base pairing energy (BPE) for the C-C+ pair is 169.7 kJ/mol, which is higher than the BPE of the canonical Watson–Crick base pair between guanine and cytosine (96.6 kJ/mol) and the neutral C-C base pair (68.0 kJ/mol) [123]. Like other nucleic acid structures, the stability of the i-motif depends on various factors, including sequence nature, temperature, and ionic strength.

Significant for understanding the factors affecting the stability of this structure are studies on chemically modified i-motif structures. Hemi-protonated C-C+ base pairs are key interactions for the stability of the i-motif. The impact of chemical modifications in these base pairs has been studied in various contexts. Wadkins et al. [124] demonstrated that cytosine modification can have varying effects on i-motif stability depending on environmental conditions. For example, replacing cytosine with its halogenated analogs, such as 5-fluoro, 5-bromo, and 5-iodocytosine, increases the stability of the i-motif under acidic conditions [125]. These modifications provide some control over the stability of the i-motif in laboratory experiments and offer insight into the structure and formation process of the i-motif.

In contrast, the Waller laboratory explored various cytosine modifications and determined that i-motifs stable at physiological pH often consisted of methylated cytosines. This discovery suggests that methylation may contribute to the formation of i-motifs in living cells [126]. Studies have also been conducted on sugar and phosphate backbone modifications. Sugar modifications generally destabilized i-motif structures. Additional substituents in the sugar ring oriented toward the compact smaller groove of the i-motif structure cause steric clashes, further destabilizing the structure [127].

The arrangement of the sugar–phosphate backbone in i-motif folding results in extremely short distances between adjacent phosphates. To mitigate repulsion between negatively charged phosphate backbones, several backbone modifications were examined. Mergny and Lacroix investigated the impact of thiophosphate and methylphosphonate backbones compared to the phosphodiester backbone. Their studies show that only backbones with phosphodiester and thiophosphate linkages allow the formation of the i-motif. They hypothesized that while the methylphosphonate backbone is neutral, the bulkiness of the methyl group prevents i-motif formation. Incorporating thiophosphates into several cytosine-rich DNA sequences leads to the formation of stable i-motif structures at neutral pH, which are only a few degrees less stable than unmodified structures [127,128].

Another backbone modification investigated involved replacing the negatively charged sugar–phosphate backbone with a neutral polyamide backbone, namely peptide nucleic acid (PNA). Balasubramanian et al. studied the effect of PNA on the model hexanucleotide sequence p(TCCCCC) using mass spectrometry with nano-electrospray ionization. It was shown that PNA forms stable i-motif structures, but the folding of the i-motif occurs in a narrower pH range (4.1–4.5) compared to its DNA counterpart (4.5–6.5) [129].

The Waller group and the Burrows group also investigated the effect of cytosine-rich DNA sequence length on the folding of intramolecular i-motif structures under physiological conditions. The formation of the i-motif was assessed using ultraviolet spectroscopy and circular dichroism. The overall conclusion from these studies is that under the same experimental conditions, the i-motif structure with a greater number of C-C+ base pairs is more stable. Researchers argue that it is possible to achieve i-motif stability at physiological pH without using modifications, provided that the minimal length of the cytosine-rich segment contains at least five contiguous cytosines [130,131].

To understand and obtain a stable i-motif structure, the effects of ionic strength and molecular crowding were also investigated. It was found that i-motif structures are influenced by the ionic strength of the solution. Mergny et al., demonstrated that increasing NaCl concentration from 0 to 100 mM destabilizes i-motif structures. Higher NaCl concentrations (300 mM) did not cause further destabilization. The same trend of decreasing i-motif stability with increasing ionic strength was observed in sequences present in the *n-MYC* gene promoter [12,127]. Regarding molecular crowding, macromolecular crowding agents, such as high-molecular-weight polyethylene glycols (PEG), are widely used to mimic the crowded environment that nucleic acids would have inside the cell. Crowding conditions preferentially stabilize both i-motif and G-quadruplex structures over duplexes and single-stranded DNA. For example, in a 1:1 mixture of guanine- and cytosine-rich sequences, molecular crowding conditions shift the equilibrium toward G-quadruplex and i-motif structures, preventing the formation of Watson–Crick duplexes [132,133].

Despite many potential factors favoring the formation of the i-motif, the acidic pH of the environment remains one of the main conditions for the creation of the i-motif. The requirement for protonation of half of the cytosines forming pairs to create the i-motif structure in vitro has led to this form of DNA having a wide range of applications in nanotechnology. During pH changes, the i-motif can reversibly fold and unfold into a hairpin-like structure, offering potential applications in nanotechnology; for example, in designing nanomachines for analytical and biomedical purposes [134,135,136,137], as switches for logical operations [138,139], and as sensors for mapping pH changes in living cells [140]. Measuring intracellular pH is a fundamental goal in biological sciences, considering the crucial impact of pH on cellular processes and the consequences of dysregulated intracellular pH in certain diseases, such as cancers [141]. These examples of i-motif structure applications highlight its potential as a building block in nanobiotechnological systems [142].

Cytosine-rich DNA sequences tend to fold into the i-motif conformation at pH from slightly acidic to nearly neutral. Since cytosine protonation is involved in the formation of the i-motif, the stability of this structure is highly dependent on pH, with i-motifs favoring a slightly acidic environment. The i-motif generally achieves maximum stability at pH 4 and 5, which promotes protonation of half of the cytosines. Further lowering the pH leads to protonation of the remaining cytosines, subsequently disrupting the i-motif structure around pH ~3 [120,143]. Due to the requirement for semi-protonated base pairs, it was believed that i-motif structures could only form at acidic pH values. However, several studies have shown that stable i-motif structures can also form at neutral pH, depending on the length of the cytosine tract, loop sequences, temperature, salt concentration, sequence length, and environmental pH [130,131]. I-motif structures are much more stable in acidic pH, due to the requirement for semi-protonated cytosine pairs, but they have also been observed at neutral pH and low temperatures (4 °C), [142] under molecular crowding conditions [144], in negative supercoiling [145], in the presence of silver or copper (I) cations [146,147], and within silica nanocanals [148].

On the other hand, the requirement for acidic pH to obtain a stable i-motif structure without environmental modifications seems to be an insurmountable barrier. It is known that dysregulated pH is an adaptive feature of most cancers, regardless of their tissue or genetic origin. In normal adult cells, intracellular pH is generally lower (around 7.2) than extracellular pH (around 7.4). However, cancer cells have higher intracellular pH (around 7.4) and lower extracellular pH (6.7–7.1). Under these conditions, cytosine-rich DNA sequences can adopt i-motif structures in vivo and modulate the formation of other nucleic acid structures. The acidic pH of the tumor microenvironment, resulting from the active metabolism of cancer cells, has increased interest in pH-responsive systems for selective delivery based on the i-motif structure, which represents an interesting pH-sensitive DNA framework [120,141,149,150].

Nevertheless, there are still limited published studies on the biological function and formation of i-motif structures and their stability in vivo. Recently, Zeraati et al. [61] managed to produce and characterize an antibody fragment (iMab) that recognizes i-motif structures with high selectivity and affinity, allowing the detection of i-motifs in the nuclei of human cells. Based on their studies, they concluded that the formation of this structure in vivo is dependent on the cell cycle and pH, and it occurs in regulatory regions of the human genome, including promoters and telomeric regions. Scientists suspect that G-quadruplex and i-motif structures may play complementary roles in gene expression regulation.

Despite significant advances in research on the structural biology of the i-motif, many aspects still require further, more detailed studies. Current research suggests that the i-motif forms transiently in the cell. However, further studies are definitely needed both in vivo and in vitro to confirm the formation of this structure at different phases of the cell cycle and to discover and explain its role in various biological processes, as the role of the i-motif seems to become increasingly important as research progresses. To facilitate research on i-motifs, both in the human genome and in biotechnological applications, more information is needed on the variables affecting their formation and which ligands may influence their stabilization. There is still much to learn about this DNA structure.

## 5. Ligands Stabilizing Four-Stranded Non-Canonical Forms of DNA

### 5.1. G-Quadruplex

Nucleic acid structures in the form of G-quadruplexes and i-motifs are being studied from various perspectives and are currently considered important players in pharmacology, biology, and medicine. These structures have the potential to be used in controlled drug delivery, for example, in new techniques for fighting cancer. Drugs targeting proteins encounter numerous similar structures on their way to the target, which is why the initially studied drugs intended to interact with G-quadruplex structures exhibited low selectivity for the target structure. This low selectivity often caused unexpected effects, leading to the halt of the drug development process. Therefore, considerable attention is now being given to the search for increasingly new ligands that interact selectively with specific DNA structures. Small molecules capable of recognizing and selectively interacting with G-quadruplexes or i-motifs have enormous therapeutic potential as tools in drug target discovery and medical diagnostics [151].

Since telomerase, a protein complex that elongates telomeric sequences, is highly active in many cancer cells, leading to the immortality of such cells and tumor formation, the formation of a G-quadruplex structure in such a telomere segment is a potential biomedical target for small molecules that inhibit this telomerase activity in cancer cells. It has been found that derivatives of 2,6-diaminoanthraquinone and telomestatin are telomerase inhibitors by inducing the formation of G-quadruplex structures, after which telomerase activity in cancer cells decreases, resulting in cell death due to aging. It is worth noting that telomestatin is a natural product isolated from the soil bacterium Streptomyces anulatus and represents the first natural telomerase inhibitor due to its ability to facilitate the formation or stabilization of G-quadruplex structures owing to structural similarity between these two structures [151,152,153]. The cationic porphyrin, TMPyP4, whose planar skeleton and cationic tendency facilitate G-quadruplex formation, has also been identified as one of the first ligands of this DNA structure [154]. These pioneering works accelerated the selective development of techniques, methods, and molecules as G-quadruplex ligands. To date, from the list of available G-quadruplex ligands, several initially discovered, such as BRACO19 [155], pyridostatin [156], Phen-DC3 [157], L1H1-7OTD [158,159], all of which have negligible binding affinity to duplex DNA, making them selective ligands for G-quadruplexes, remain indispensable in biochemical, biophysical, and chemical biology studies of G-quadruplexes. The structural formulas of a few examples of G-quadruplex stabilizing ligands are shown in Table 1. To date, over 800 types of ligands of this non-canonical DNA structure have been described [71,160].

Beyond the G-quadruplex structures and their ligands in the telomeric DNA fragments, ligands and sequences forming G-quadruplexes observed in the promoters of cancer-related genes have also gained significant attention as potential biomedical targets in cancer therapy [161,162]. Quarfloxin, a G-quadruplex-interacting ligand, completed phase II clinical trials as a therapeutic candidate against various cancers, including neuroendocrine tumors, carcinoids, and lymphomas. It is believed that quarfloxin disrupts the G-quadruplex–nucleolin complexes of ribosomal DNA in the nucleolus, which in turn causes the redistribution of nucleolin to the nucleoplasm, where it binds to the G-quadruplex in the promoter region of the proto-oncogene *c-MYC* to inhibit gene expression.

G-quadruplex-interacting ligands can contribute to suppressing further *c-MYC* expression by stabilizing the G-quadruplex via the ligand. In this context, G-quadruplex-interacting ligands targeting *c-MYC* have been studied over the past two decades for their potential use in cancer therapy. Phase III trials of quarfloxin are currently not being conducted due to high albumin binding [151]. Dash and colleagues reported that the moon-shaped thiazole peptide, TH3, preferentially stabilizes the *c-MYC* G-quadruplex compared to the G-quadruplex structure in other promoters, making it a specific ligand [163]. Additionally, other cancer-related genes, including *hTERT* [164], *c-kit* [165], *kRAS* [166], and *BCL2* [167], have been identified as genes where G-quadruplex formation is involved in transcriptional regulation, and its stabilization by ligands attenuated promoter activity, ultimately inducing tumor apoptosis.

Extensive research on G-quadruplex ligands leads to the belief that G-quadruplexes are capable of forming abundantly in guanine-rich regions of DNA and RNA. Although numerous researchers have made significant efforts to obtain highly active G-quadruplex ligands, and some have achieved great success in developing drugs utilizing these ligands in vivo, these drugs are still only halfway to being approved for clinical use. One of the obstacles hindering the clinical application of specific molecules interacting with G-quadruplex structures is the required selectivity [151,168]. Therefore, research continues today on designing G-quadruplex ligands and their application in developing anticancer and antiviral therapies, as well as identifying G-quadruplexes in living cells. As long as new ligands, technologies, and theories are developed, the G-quadruplex structure will have broad and significant biomedical applications [107].

Minor groove-binding drugs such as netropsin and Hoechst 33258 have shown significant potential in stabilizing G-quadruplex–duplex hybrids (QDH). These hybrids, which include a hairpin duplex within the G-quadruplex core, demonstrate sequence-specific recognition facilitated by these drugs. The binding of netropsin and Hoechst 33258 significantly enhances the thermal stability of QDH structures, emphasizing the crucial role of the interaction between the stem-loop and the G-quadruplex core in maintaining structural integrity [169]. Photosensitive ligands like DTE and TMPyP4 have been studied for their ability to manipulate human telomeric G-quadruplexes under visible light. These ligands display distinct responses to light, affecting the thermal unfolding pathways of G4 structures. Their ability to induce multi-step melting pathways and stabilize G4 under light exposure underscores their potential in photopharmacological applications [170]. Recent research has highlighted the efficacy of a potent guanidine derivative in stabilizing the human *BCL-2* G-quadruplex DNA. This compound selectively binds to the G-quadruplex, resulting in significant stabilization and subsequent downregulation of *BCL-2* transcription. Such findings illustrate the therapeutic potential of guanidine derivatives in targeting oncogenes frequently mutated in cancer [171]. Pyridostatin is a well-known ligand for stabilizing G-quadruplexes. Studies have demonstrated its strong binding affinity and stabilization effect on the G-quadruplex structure within the *PARP1* gene promoter region. Other G-quadruplex binders have been analyzed using techniques like NMR, CD, and fluorescence titration, showing varying degrees of stabilization and binding modes. Pyridostatin, however, remains one of the most effective stabilizers identified [172].

A novel class of multitarget-directed ligands has been designed to stabilize G-quadruplex structures and inhibit human carbonic anhydrases IX and XII. These ligands, based on a berberine scaffold, demonstrate dual functionality by stabilizing G-quadruplexes and exhibiting cytotoxic effects against cancer cells. This dual action makes them promising candidates for anticancer therapies [173]. New synthetic ligands, such as TPB3P and TPB3Py, have shown high stabilization effects and selectivity for G4 over duplex DNAs. These compounds, featuring polyamine pendant arms and central aromatic cores, exhibit strong cytotoxicity in various cancer cell lines. Their encapsulation in liposomes and targeting with AS1411 aptamers further enhance their effectiveness, achieving nanomolar IC50 values [174].

The ability of small molecule ligands to distinguish between DNA and RNA G-quadruplexes has been extensively studied due to the structural differences between these two types of G4s and their implications in biological processes. DNA and RNA G-quadruplexes share a common structure of stacked guanine tetrads, but they differ significantly in terms of their topology, stability, and conformation. DNA G4s can adopt multiple topologies such as parallel, antiparallel, or hybrid forms, depending on the sequence and environmental conditions. These structures are typically less stable than RNA G4s due to the absence of the 2′-OH group in deoxyribose. RNA G4s almost exclusively form parallel topologies due to the presence of the ribose 2′-OH group, which constrains the conformation of RNA, making it more rigid and thermodynamically stable than DNA G4s [96,175].

Several small molecules (e.g., berberine, quarfloxin, PhenDC3, RHPS4, BRACO-19, telomestatin, pyridostatin, and carboxypyridostatin) have been designed to target G4s in both DNA and RNA. These molecules typically interact with the G-tetrads via π-π stacking or with the loops and grooves via hydrogen bonding and electrostatic interactions [176]. Pyridostatin is a well-known G4 stabilizer that has been used in various studies to detect G4 formation in both DNA and RNA. It was initially designed to target DNA G4s and has shown strong binding to telomeric DNA G4s, inhibiting telomerase activity [175,177]. Pyridostatin has also been reported to bind more strongly to RNA G4s, suggesting that it may distinguish between the two forms [178]. It was demonstrated [179] that both PhenDC3 and pyridostatin can act as molecular chaperones and promote the formation of RNA G4s in vitro, while BRACO19 interacts mostly with folded G4s. While it has been tested primarily on DNA, its interaction with RNA G4s appears to be limited, making it more selective for DNA G4s. NaphthoTASQ is a synthetic molecule designed to target G4 structures in both DNA and RNA. Studies have shown that NaphthoTASQ binds to both DNA and RNA G4s, though its efficiency and selectivity depend on the context in which it is used. It allowed for tracking both DNA and RNA G4s in fixed cells and RNA G4s in living cells [177,180]. However, the selectivity of a ligand for G4 DNA over G4 RNA, and even for a single G-quadruplex-forming sequence, is the greatest challenge in the field of G4 ligands [178].

The identification of ligands that stabilize G-quadruplexes has advanced our understanding of G4 biology and its potential therapeutic applications. Minor groove-binding drugs, photosensitive ligands, guanidine derivatives, pyridostatin, multitarget-directed ligands, and novel synthetic compounds have all demonstrated significant stabilization effects on G-quadruplex structures. These ligands not only provide insights into the structural dynamics of G-quadruplexes but also offer promising avenues for the development of new cancer therapies and molecular probes.

### 5.2. i-Motif

When it comes to ligands that selectively interact with i-motif DNA structures, both the efficacy and the number of such ligands are significantly less researched and fewer compared to those for G-quadruplex structures. Developing selective i-motif ligands using conventional methods is challenging due to the structural complexity of the i-motif and its preferential formation under acidic pH conditions. The first example of an i-motif binding compound was published by Fedoroff et al. [181] in 2000. Using human telomeric sequences d(CCCAAT)_4_ and d(AATCCC)_4_, they investigated the binding properties of the cationic porphyrin TMPyP4, which was found to bind to the given nucleotide sequence and promote i-motif formation at pH 4.5. However, this ligand only served as a clue for further development efforts to improve the selectivity of subsequent i-motif ligands, as it also facilitates G-quadruplex formation, thus disqualifying it as a selective ligand for a specific DNA form [181].

Other ligands described and studied for their selectivity toward i-motif structures include phenanthroline derivatives [182], neomycin–perylene conjugates [183], crystal violet [184], thioflavin T [185], and berberine [186], but these ligands also do not exhibit significant selectivity for i-motif structures compared to duplex and G-quadruplex structures. In subsequent years, Hurley and colleagues identified a cholestane derivative as a strong and specific i-motif binding compound, which may provide an approach to regulate *BCL2* transcription in cancer cells [187]. Currently, only a few specific i-motif ligands, such as the type II topoisomerase inhibitor mitoxantrone [188], peptidomimetic ligands [57], benzothiophene derivatives [189], and acridone derivatives [190], have been tested in cellular systems. The structural formulas of a few i-motif stabilizing ligands are shown in Table 2. Dzatko et al. [191] conducted experiments in cells using NMR to determine whether i-motifs remain stable in the complex cellular environment of living mammalian cells. Interest in the therapeutic potential of i-motif structures increased after the discovery of their in vivo existence in the nuclei of human cells by the group of Christ and Dinger. This was made possible by the discovery of an antibody capable of specifically binding the i-motif in the cell nucleus of living organisms. These studies confirmed that the i-motif can exist in regulatory regions of the genome of living cells under physiological conditions [61]. However, to this day, the development of selective ligands for the i-motif is difficult because the i-motif has a similar four-stranded structural topology to G-quadruplexes.

An interesting observation in the search for i-motif ligands was described by Chen et al. [192], who discovered that carboxylated single-walled carbon nanotubes can be considered leading candidates for the first ligand capable of selectively stabilizing human telomeric i-motif DNA. The mechanism of this process, as proposed by Chen et al. [192], involved several rather complex biological mechanisms, ultimately leading to the generation of a DNA damage response at the telomeric level. The researchers observed subsequent inhibition of telomerase activity in the studied living cells. They suggested that stabilization of the i-motif structure and the accompanying formation of G-quadruplexes lead to the unmasking of telomeres and the relocation of telomere-binding proteins, generating a DNA damage response at the telomeric level and subsequently halting the growth of cancer cells. The possibility of selectively inducing the formation of the telomeric i-motif by carboxylated single-walled carbon nanotubes was first reported by Li et al. [193] These scientists discovered that single-walled carbon nanotubes inhibit duplex DNA association and bind to the major groove at the 5′ end strand at neutral or even slightly alkaline pH = 8 [194]. The binding is stabilized, among other things, by electrostatic interactions between the carboxyl-modified nanotube and the C:C+ base pairs. However, under these conditions, semi-protonated cytosine pairs should not exist. Another hypothesis proposed by Li et al. [193] is that the nanotubes serve as condensation nuclei to increase the tendency for DNA aggregation, and this effect should facilitate C:C+ protonation. Another interesting observation was that non-carboxylated nanotubes can induce i-motif formation at neutral pH under molecular crowding conditions induced by the presence of polyethylene glycol. Interestingly, these crowding conditions could not induce i-motif formation without the presence of nanotubes [195]. This suggests that i-motif formation is facilitated by the specific shape of the nanotubes, not their charge or attached functional group. However, detailed reports on the specific mechanism of interaction between systems composed of telomeric DNA containing non-canonical DNA forms such as i-motifs or G-quadruplexes and carbon nanotubes are lacking in the available literature. These interactions are still not fully understood. Much attention must be devoted to elucidating these molecular interaction mechanisms, the induced biological effects, and long-term biological safety. This will help direct proper methods and applications of appropriate systems in various circumstances.

Despite discoveries related to i-motif ligands, the number of known specific substances binding to this DNA form is very limited compared to G-quadruplex ligands. For many years, the G-quadruplex structure has garnered significant research interest, primarily due to its thermodynamic stability under physiological conditions. Yet, research in recent years provides further important information that sheds new light on this DNA structure, allowing the biological role of the i-motif structure, its interactions with nanotubes, and the interest in this structure to flourish in the coming years [148,196]. To date, although several promising applications have been designed based on the specific interactions of ligands with DNA, few applications can be used in vivo. Many interactions between ligands and DNA have been studied and visualized in vitro. It must be verified whether they can work in vivo. The information gained will help find new, further applications of nanotube connections with DNA structures, including the i-motif structure, in gene therapies, drug delivery, and nanotechnology [13,197].

In recent years, significant progress has been made in the discovery and study of ligands that target G-quadruplexes, resulting in a plethora of new ligands, research methodologies, and activity data. Similarly, i-motifs have garnered substantial research interest. Addressing the current need to index and organize these valuable resources, Wang et al. (2022) [198] have developed the G-quadruplex and i-motif ligand database, known as G4LDB. This database offers a comprehensive collection of small molecular ligands for both G-quadruplexes and i-motifs, complete with detailed physical and chemical information as well as data on their biological activities. Additionally, G4LDB features an online ligand design module that allows for the prediction of ligand binding affinity and real-time ligand–receptor docking.

## 6. Computer Simulations of Four-Stranded Non-Canonical DNA Forms

### 6.1. G-Quadruplexes

Computer simulations are indispensable for understanding the atomic-scale interactions and mechanisms that govern the formation and stability of G-quadruplexes. These nucleic acid structures, characterized by stacked guanine tetrads, are crucial in various biological processes, including the maintenance of telomeres and the regulation of oncogene promoters. While traditional experimental techniques such as X-ray crystallography and nuclear magnetic resonance (NMR) spectroscopy provide valuable structural information, they are limited in capturing the dynamic behavior and conformational flexibility of G-quadruplexes. Molecular dynamics (MD) simulations and quantum mechanical (QM) calculations bridge this gap, offering detailed insights into the dynamic properties and energetics of G-quadruplexes. Simulations can fill gaps in experimental data, providing a more complete understanding of nucleic acid structures and their interactions. They help interpret experimental results and offer predictions about molecular behaviors that are challenging to observe experimentally.

MD simulations enable researchers to explore the time evolution of G-quadruplex structures under various environmental conditions, shedding light on their stability, folding pathways, and interactions with ligands at an atomic level [199,200]. These simulations replicate the dynamic behavior of G-quadruplexes over time, providing a more comprehensive understanding than static experimental methods. Additionally, QM calculations elucidate the energetic landscapes and electronic properties of these structures, which is crucial for the design of selective and effective G-quadruplex-stabilizing ligands [201]. MD simulations have been applied to a wide range of nucleic acid systems, from small tetranucleotides to large complexes such as the ribosome. This versatility makes MD a valuable tool for studying diverse nucleic acid structures, including G-quadruplexes [202].

The reliability of MD simulations is heavily dependent on the accuracy of the molecular mechanics force fields (FFs) used [203]. Current FFs may not perfectly capture all the intricate interactions within nucleic acids, leading to potential inaccuracies in the simulation results. Additionally, simulations are typically limited to relatively short time scales (nanoseconds to microseconds) compared to the actual biological processes (which can span milliseconds to seconds). This can restrict the observation of long-term behaviors and slow conformational changes. Moreover, the quality of the initial structure used in the simulation is critical. Inaccuracies in the starting geometry, such as incorrect placement of ions or improper conformations, can bias the simulation outcomes. The simulation time may also be insufficient to correct these inaccuracies. Despite these limitations, molecular dynamics simulations remain a powerful and increasingly sophisticated tool for studying the structural dynamics of nucleic acids. The ability to visualize and analyze molecular motions at an atomic level provides invaluable insights into the behavior of complex nucleic acid structures like G-quadruplexes [202].

The simulation of G-quadruplexes has seen significant methodological advancements over the past few decades. Early studies primarily utilized basic MD simulations to investigate the stability and conformational preferences of simple G-quadruplex models. These initial efforts employed classical force fields such as AMBER to simulate the dynamics of G-quadruplexes and understand their interactions with monovalent cations like potassium and sodium [204]. As computational power and methodologies evolved, more sophisticated techniques integrating QM calculations and enhanced sampling methods, such as steered molecular dynamics, replica exchange, and metadynamics, were developed.

Clay and Gould [201] combined QM and molecular mechanics (MM) simulations to investigate the stability of human telomeric G-quadruplexes, highlighting significant structural differences when potassium ions were replaced with sodium ions. Šponer and Špačková [205] reviewed advanced MD simulations to study four-stranded DNA structures, providing detailed insights into their dynamic behavior and stability. Their work demonstrated the importance of ion presence and type, loop lengths, and sequence composition in the stability and formation of G-quadruplexes.

**Figure 8:**
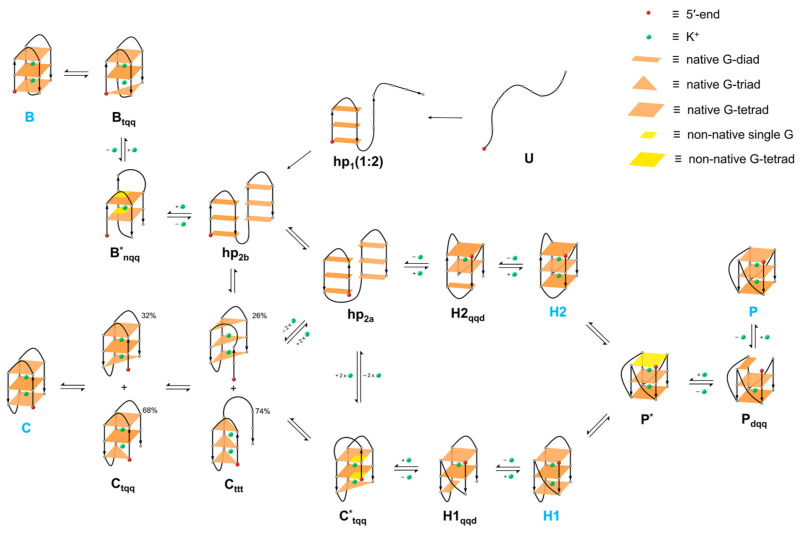
G-quadruplex folding model proposed in the study by Kim et al. [213] This model accounts for all possible folding pathways, viz. chair (C), basket (B), hybrids (H1 and H2), and propeller (P), and explicitly shows their interconversion mechanisms. G-stacking in each layer is classified as diad (d), triad (t), tetrad (q), or none (n) depending on the number status of guanine-aggregation through hydrogen bonds. Thus, three consecutive letters in subscripts in the generic notation indicate G-aggregation numbers pertaining to three layers of G-stacking in the G1 to G3 direction. Superscript “*” indicates the existence of non-native G-pairing. A single-hairpin is denoted as “hp1” and a double-hairpin as hp2a or hp2b. (Reprinted with permission from Ref. [213] Copyright 2023 American Chemical Society).

The formation and stability of G-quadruplexes are influenced by several factors, including the presence of specific ions, loop lengths, and sequence composition. The G-tetrad core’s stacking interactions, mediated by Hoogsteen hydrogen bonds and stabilized by monovalent cations (primarily potassium), are critical for the structural integrity of G-quadruplexes. MD simulations have demonstrated that the arrangement of these tetrads and the nature of the intervening loops significantly affect the folding and stability of the G-quadruplex. Key mechanisms of G-quadruplex formation and stabilization uncovered by computer simulations include:

- *Ion Coordination and Stabilization*: The role of monovalent cations (K^+^ and Na^+^) is pivotal in stabilizing the G-quadruplex structures. Simulations have shown that potassium ions, due to their size and coordination properties, fit perfectly within the central channel of G-quadruplexes, stabilizing the G-tetrads through electrostatic interactions [206,207]. Sodium ions, while also stabilizing, do so less effectively compared to potassium, often leading to different conformational preferences in the G-quadruplex structure [201,208,209]. Kinetic analysis based on Markov modeling showed that presence of Na^+^ modestly enhances an antiparallel G-quadruplex topology, while K^+^ drives G-quadruplex into a parallel/hybrid topology with much higher affinity than Na^+^ does [210].
- *Folding Pathways and Kinetics*: Simulations have provided insights into the folding pathways of G-quadruplexes, revealing multiple intermediate states that the DNA strands can adopt before forming the stable G-quadruplex structure. In general, the folding of G-quadruplexes is best described by a kinetic partitioning (KP) mechanism. KP involves competition between at least two (and often many) well-separated and structurally distinct conformational ensembles. The KP folding landscape contrasts with the funneled landscape, containing deep competing free-energy minima (alternative folds or conformational basins) separated by large free-energy barriers. Only a fraction of molecules fold directly to the native basin, which is most populated at thermodynamic equilibrium. Other molecules initially fold into competing (non-native) basins, becoming trapped in different basins. Thermodynamic equilibrium is reached after numerous misfolding–unfolding events, leading to the equilibrium population of all basins. Therefore, the whole process is slow. Human telomeric G-quadruplex sequences can exhibit multiple folds at thermodynamic equilibrium, with other basins transiently populated during folding. The relative stabilities of different basins can be significantly influenced by the environment [211]. The MD simulations indicate that the immense complexity of the G-quadruplex folding landscape is linked to the ability of many G-quadruplex-folding sequences to adopt multiple alternative structures with different patterns of anti and syn guanosines, which, once formed, have long lifetimes. If these structures appear during folding but are absent in the final thermodynamic equilibrium, detecting and structurally resolving them becomes very challenging [211,212,213]. Bian et al. [214] employed a hybrid atomistic structure-based model to investigate the folding dynamics of the human telomeric DNA G-quadruplex. This model integrates structural information from three known G-quadruplex topologies: hybrid 1, hybrid 2, and chair-type conformations. The model was validated by its ability to replicate experimental observations, specifically that the hybrid-1 conformation is the major fold while hybrid 2 is more kinetically accessible. A three-step mechanism was identified for the formation of the hybrid 1 conformation, whereas the hybrid 2 and chair-type conformations followed a two-step mechanism. The presence of inappropriate syn/anti guanine nucleotide combinations was found to slow down the folding process significantly. In a recent study, Kim et al. [213] proposed a folding scheme for the human telomeric G-quadruplex using state-of-the-art enhanced sampling molecular dynamics simulations at the all-atom level. As illustrated in Figure 8, the G-quadruplex folding process begins with the formation of a single-hairpin structure, followed by the formation of double hairpins. These double hairpins then proceed along distinct folding pathways, leading to various G-quadruplex topologies, including antiparallel chair, antiparallel basket, hybrids 1 and 2, and parallel propeller forms. Additionally, three-triad and two-tetrad structures with antiparallel backbone alignment act as crucial intermediates, facilitating the folding process and transitions between different G-quadruplex structures. This computational study also demonstrated that the structural ensemble and ion capture by human telomeric DNA dramatically respond to temperature increases.

- *Loop Dynamics and Conformational Flexibility*: The dynamics and flexibility of the loops connecting the G-tetrads are crucial for the overall stability and folding of G-quadruplexes [215]. Simulations have shown that loop length and sequence composition can dramatically affect the folding kinetics and stability of the resulting structure [216]. The simulation results by Islam et al. [217] suggest that the loops may exist as a dynamic continuum of interconverting substates, which would be difficult to fully capture by available experimental methods. Additionally, they demonstrated that long simulations are needed to sufficiently exhaustively characterize quadruplex DNA loop dynamics without visible bias from the starting structure. One of the most interesting structural observations was the end capping of a quadruplex with the terminal adenine base. Markov-state modeling was employed to understand the trends of structural transitions in the propeller loops. Transition rates estimated by transition path theory indicated that loop interconversions occur on microsecond to dozens of microseconds time scales. Using the bsc0 AMBER force field, simulations visualized all the main conformational substates on the landscape of the TTA propeller loops [218]. Studies of decomposition thermodynamics indicated that the G-tetrad is strongly stabilized by interactions involving the sugar–phosphate backbone and TTA loops. The energetics of guanine association alone is not the decisive factor [219].
- *Hydration and Solvent Effects*: The role of water molecules and hydration shells around G-quadruplexes has been extensively studied using MD simulations. These studies have highlighted the importance of water-mediated interactions in stabilizing the G-tetrads and influencing the overall conformation of the G-quadruplex. Chowdhury and Bansal found that the guanine quadruplex is stable, even in the absence of coordinated cations. Water molecules can occupy the empty coordination sites in this situation. Sodium ions can enter a preformed quadruplex through the ends and travel within the quadruplex channel without significantly distorting the G-tetrad geometry. Meanwhile, water molecules can exit the channel through the ends as well as through the grooves [206]. Additionally, the presence of water molecules is essential for the accurate representation of the folding landscapes of G-quadruplexes in simulations. Hydration shells around the DNA provide a realistic environment that affects the energy barriers and the pathways of folding and unfolding processes. Long simulations are necessary to capture the exhaustive dynamics of these hydration effects without bias from the starting structures [217]. These findings underscore the intricate interplay between G-quadruplexes and their solvent environment, highlighting the necessity of considering solvent effects in computational studies of these structures. Understanding these interactions is crucial for accurate modeling of G-quadruplex stability, folding mechanisms, and their biological functions [220].
- *Ligands Stabilizing G-quadruplexes:* Identifying and designing ligands that selectively bind and stabilize G-quadruplexes is of great interest, particularly for therapeutic applications in oncology. Ligands such as telomestatin and various small molecules have been shown to preferentially stabilize G-quadruplex structures over duplex DNA, thereby inhibiting the activity of telomerase and certain oncogenes. Docking studies, ligand-based methods, especially QSAR (Quantitative Structure–Activity Relationships), pharmacophore models, and MD simulations have been pivotal in understanding the binding modes and affinities of these ligands [221,222]. For instance, Ramos et al. [200] investigated a diketopyrrolo[3,4-c]pyrrole derivative and found that it binds to G-quadruplexes through various modes, significantly stabilizing these structures and inhibiting oncogene promoter activity. Mulliri et al. [223] studied substituted pyrazolo[1,2-a]benzo[1,2,3,4]tetrazine-3-one derivatives as G-quadruplex stabilizing/destabilizing ligands. The MD results in this study were particularly important when considering that the docking study indicated that both the stabilizing and destabilizing compounds display a similar negative binding free energy, while the MD simulations discriminated the stabilizing/destabilizing activity of the ligands.

G-quadruplex stabilizing ligands share many characteristics, including polycyclic aromatic scaffolding. These ligands interact with the G-quadruplex according to *π*–*π* stacking with the terminal tetrad, and the lateral positively charged moieties interact with the phosphate groups within the loops. They also have a propensity to bind to the upper and lower end positions of the G-tetrads [224]. Indeed, simulations have revealed that ligand binding often involves stacking interactions with the terminal G-tetrads and electrostatic interactions with the loops and grooves of the G-quadruplex [225]. These interactions are crucial for enhancing the thermal stability of the G-quadruplex and preventing its unwinding.

Computer simulations have significantly advanced our understanding of G-quadruplexes by providing detailed insights into their formation, stability, and interactions with ligands. MD and QM simulations complement experimental techniques, offering dynamic and energetic perspectives essential for the rational design of G-quadruplex-targeting drugs. The continuous development of computational methods promises to further elucidate the complex behaviors of these biologically significant structures, paving the way for novel therapeutic strategies.

### 6.2. i-Motifs

I-motif structures, which are four-stranded DNA formations stabilized by cytosine-cytosine+ base pairing, have attracted significant attention due to their potential roles in biological processes and applications in nanotechnology. Molecular dynamics simulations have provided substantial insights into the mechanisms of formation and stability of i-motif structures.

It is generally known that the main factor responsible for the stability of the i-motif structure is the Hoogsteen hydrogen bonds between C:C+ pairs. MD simulations have shown that the deprotonated telomeric i-motif structure is not stable at room temperature, which agrees with experimental studies [226]. It was also found that very long, 230nm sequences, forming nanowires based on TC5 sequences, deteriorate quickly upon losing protonated cytosine in the tetrad [227].

Smiatek and Heuer proposed an unfolding mechanism in which the release of two protons is enough to cause the telomeric i-motif to unfold into hairpin configurations as well as fully unfolded structures. Furthermore, the hairpin conformation unfolds into random coil stretched configurations, where additional proton–DNA contacts do not necessarily need to be broken, meaning that the protons remain attached to the DNA strand [228].

Studies of i-motif stability in the presence of ionic liquids showed that the i-motif formed even at physiological pH in the choline dhp-containing solution. Moreover, its thermodynamic stability was greater than that of G-quadruplex [229]. Another interesting application of MD simulations was generation of samples of the gas-phase conformations for trapped ion mass spectrometry for analysis candidate structures during i-motif unfolding [230].

MD studies of two topologies of the intermolecular i-motif have shown the importance of the interaction of the phosphodiester backbones through the narrow groove. This emphasizes the significance of the sugar–sugar contacts across the narrow groove, which, by enforcing the optimal backbone twisting, are essential for base stacking and the overall stability of the i-motif [122].

Protopopova et al. [231] utilized MD simulations to investigate the formation of i-motif structures from C-rich oligonucleotides. They determined a number of bases that can form a loop over a major groove. They built two monomers with three pairs of cytidines in the i-motif core and two or three cytidines in the major loops. The structure with three cytidines was stable while the structure with two cytidines in the loops was unstable.

Panczyk and Wolski [232] performed MD simulations to analyze the stability of the telomeric i-motif and its corresponding Watson–Crick duplex under varying pH and temperature conditions. They demonstrated that the protonation state of cytosines plays a crucial role in stabilizing the i-motif and destabilizing the Watson–Crick duplex. At acidic pH, the i-motif remains stable due to the formation of strong hydrogen bonds. However, deprotonation leads to spontaneous unfolding of the i-motif into a hairpin structure.

Mondal et al. [233] performed comprehensive molecular dynamics and quantum chemical studies aimed at understanding the structural properties, the influence of solvent, and the interplay among the intramolecular interactions in i-motif DNA. They considered two different topologies, 3′E and 5′E, analyzing them in two cases: at neutral pH with normal unprotonated cytosines and at acidic pH when half of the cytosines were protonated. They investigated the energetic preference of the stacking geometry of intercalated C:C+ pairs in the i-motif core using molecular modeling and quantum chemical calculations. They also studied the equilibrium dynamics of the i-motif, conformational properties, stacking interactions, energetic preference of the base pair stacking geometry, and the role of solvent. They found that the conformational dynamics of the i-motif is mainly associated with its loop motion. The unfolding of the i-motif is related to the loss of water molecules interacting with the N4 atom of cytosine along the wide grooves and the disruption of backbone interactions along the narrow grooves.

**Figure 9:**
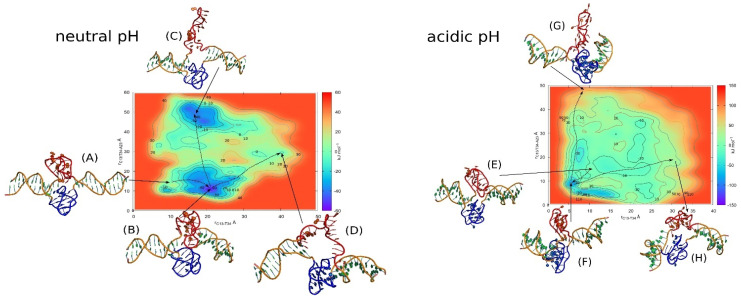
Contour maps of the potential of mean force (PMF) accompanied the biased unfolding of i-motifs within the i-motif+G-quadruplex structure at neutral pH, i.e., with the unprotonated i-motif. The collective variables *r*C13-T34 and *r*C13/T34-A23 are defined as distances between centers of masses of C13 and T34 bases and C13 and T34 taken together and A23, respectively. State A is the initial configuration, which is reference point (zero) in the PMF map. State B is the lowest energy configuration found, while states C and D are unfolded states close to the hairpin (C) or the random coil (D) configuration of the i-motif. (E)–(H) Contour maps of the potential of mean force (PMF) accompanied the biased unfolding of i-motifs within the i-motif+G-quadruplex structure at acidic pH, i.e., with the protonated i-motif (Adapted with permission from Ref. [234], Copyright 2019 American Chemical Society).

Wolski et al. [234] studied the effect of the presence of C-rich single strands attached to the terminal parts of the i-motif structure and the presence of a complementary G-quadruplex formed on the guanine-rich strand. They found that the G-quadruplex within the complementary G-rich strand enhances the stability of the i-motif at both acidic and neutral pH. They also observed another stable secondary structure within the C-rich strand, which is the hairpin structure at neutral pH. Figure 9 shows the free energy maps obtained for these cases using the metadynamics enhanced sampling method.

Wolski, Nieszporek, and Panczyk [150,235] explored the interaction of i-motif-forming DNA with carbon nanotubes (CNTs) for potential applications in drug delivery systems. Their MD simulations indicated that the DNA-CNT constructs could effectively modulate the release of doxorubicin in response to pH changes. The folding and unfolding of the i-motif structure control the release mechanism, demonstrating the stability of i-motifs in varying environmental conditions.

Recently, Shamim et al. [236] and Xi et al. [237] showed that the loop region may be the most important factor affecting the structural stability of i-motifs. They built several model i-motifs with different loop lengths and investigated the coefficient of determination between the thermal stability determined by experimental methods and loop flexibility estimated by MD simulation using model i-motifs.

Lopez et al. [238] examined the adsorption behavior of poly-cytosine DNA on graphene oxide surfaces using MD simulations. They found that the adsorption strength is significantly influenced by pH, with stronger interactions at neutral pH due to the flexible phosphate backbone of the i-motif. This flexibility allows for more robust hydrogen bonding with the graphene oxide surface, highlighting the importance of environmental conditions in the stability of i-motif structures.

Molecular modeling has been applied to study an intriguing observation related to the interaction of carboxylated carbon nanotubes with i-motif-forming sequences. As already mentioned [192], the suppression of telomerase activity was attributed to i-motif formation within the telomeres due to interaction with carboxylated carbon nanotubes. Wojton et al. [239] recently performed comprehensive theoretical studies aimed at understanding the molecular mechanism of i-motif stabilization by carboxylated carbon nanotubes. They concluded that the most likely reason is the local reduction in pH by such nanotubes, as direct routes of proton transfer from carboxyl groups to cytosines were found to be highly energetically unfavorable.

It is worth mentioning that, as found by Panczyk et al. [240], both i-motif and G-quadruplex structures are stable in a molecular mechanics sense only when using well-tuned atomistic force fields for nucleic acid simulations. Coarse-grained force fields, which are very useful in many cases due to the enormous acceleration of structural rearrangements in macromolecules, are not applicable for describing non-canonical DNA forms. None of the tested coarse-grained force fields allowed the i-motif or G-quadruplex structures to remain stable.

The insights gained from these MD simulations have profound implications for the use of i-motif structures in nanotechnology and medicine. The ability to control the stability and formation of i-motifs through environmental factors such as pH and temperature makes them ideal candidates for targeted drug delivery systems and the design of nanoscale materials with specific mechanical properties.

## 7. Summary

This review has explored the structural intricacies, stability factors, and potential applications of non-canonical DNA structures, particularly focusing on i-motifs and G-quadruplexes. G-quadruplexes are four-stranded structures formed by guanine-rich sequences that can adopt various topologies, including parallel, antiparallel, and hybrid forms. The formation of G-quadruplexes is stabilized by Hoogsteen hydrogen bonds and monovalent cations like potassium. In contrast, i-motifs are four-stranded DNA structures formed by cytosine-rich sequences under acidic conditions, stabilized by hemiprotonated cytosine–cytosine (C:C+) base pairs.

The stability of G-quadruplexes and i-motifs is influenced by factors such as sequence composition, the presence of specific ions, and environmental conditions like pH for i-motifs. The dynamic nature of these structures, including their folding and unfolding mechanisms, is crucial for their biological functions and is often studied using techniques such as molecular dynamics simulations. G-quadruplexes are found in key genomic regions, such as telomeres and promoter regions of oncogenes, playing roles in the regulation of gene expression, maintenance of genome stability, and cellular aging. Although less prevalent, i-motifs are also implicated in gene regulation and are suggested to have functional roles in the genome, especially in the regulation of transcription.

The unique properties of G-quadruplexes and i-motifs make them attractive targets for drug design. Small molecules that can selectively bind and stabilize these structures have potential therapeutic applications, particularly in cancer treatment. Additionally, G-quadruplexes and i-motifs are being explored for their use in nanotechnology and molecular devices due to their distinct structural features and responsiveness to environmental changes.

Recent advances in high-resolution structural techniques and computational modeling have significantly enhanced our understanding of these non-canonical DNA structures. However, challenges remain, particularly in observing these structures in vivo and understanding their full range of biological functions. Despite these challenges, the ongoing research continues to uncover new aspects of these fascinating DNA structures, promising exciting developments in both basic science and applied fields.

In conclusion, this review emphasizes the importance of G-quadruplexes and i-motifs in genomic regulation and their potential as therapeutic targets and functional elements in nanotechnology. The ongoing research in this area continues to reveal new insights, highlighting the significance and future prospects of these intriguing DNA structures.

## Figures and Tables

**Table 1:** A few examples of G-quadruplex-interacting ligands and their structural formulas.

BRACO19 [155]	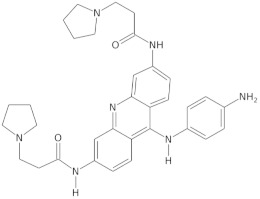
Pyridostatin [156]	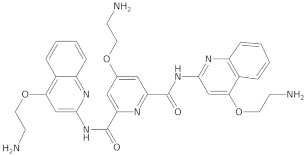
Phen-DC3 [157]	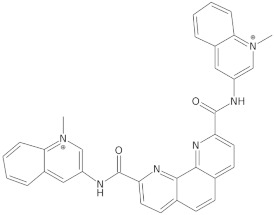
L1H1-7OTD [158,159]	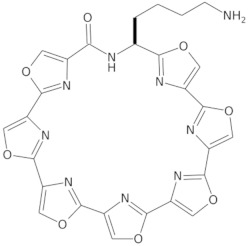

**Table 2:** A few examples of i-motif-interacting ligands and their structural formulas.

Cholestane derivative, NSC 138948 [187]	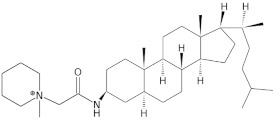
Mitoxantrone [188]	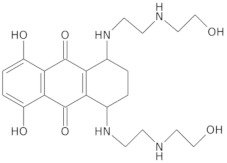
Bisprolinamide derivative, PBP1 [57]	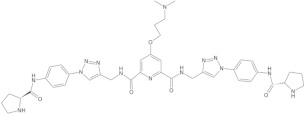
Acridone derivative, B19 [190]	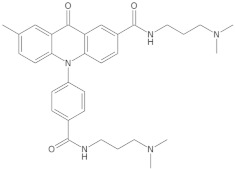

## Data Availability

Dataset available on request from the authors.
